# Firing up Cold Tumors—Targeting the Epigenetic Machinery to Enhance Cancer Immunotherapy

**DOI:** 10.3390/epigenomes5020011

**Published:** 2021-05-03

**Authors:** Guan-Ling Lin, Leah H. J. Tsai, Peter J. K. Kuppen, Michael W. Y. Chan

**Affiliations:** 1 Department of Biomedical Sciences, National Chung Cheng University, Min-Hsiung, Chia-Yi 62102, Taiwan; guan0223@gmail.com (G.-L.L.); leahtsai@alum.ccu.edu.tw (L.H.J.T.); 2 Epigenomics and Human Disease Research Center, National Chung Cheng University, Min-Hsiung, Chia-Yi 62102, Taiwan; 3 Department of Surgical Oncology, Leiden University Medical Centre, 2333 ZA Leiden, The Netherlands

Cancer immunotherapy using monoclonal antibodies targeting immune checkpoint proteins, such as PD-L1 or PD-1 (i.e., immune checkpoint blockade, ICB), have demonstrated improved overall survival in several human cancers. For example, a recent phase III clinical trial (JAVELIN Bladder 100) demonstrated that bladder cancer patients treated with Avelumab (anti-PD-L1 antibody) have significant improvement in both overall survival and progression-free survival as compared to a control group [1]. However, tumors that suppress T cell infiltration (known as cold tumor) may be resistant to ICB. Therefore, understanding the molecular mechanism leading to tumor immunoediting (selective outgrowth of variants of tumor cells able to escape immunosurveillance) and the restoration of an inflammatory tumor microenvironment (i.e., hot tumor) is crucial for the development of novel therapeutics to increase the efficacy of ICB against human cancer (Figure 1).

Recently, studies have suggested that cancer growth reprograms the epigenome, which may lead to escape from immunosurveillance. For example, epigenetic modifications, namely DNA methylation and histon modification H3K27me3, have been shown to suppress the transcription of TH1-type trafficking chemokines CXCL9 and CXCL10 in human cancer [2,3,4]. Epigenetic silencing of CXCL9/10 by promoter methylation and H3K27me3 in tumor cells thus hinders the infiltration of cytotoxic T cells for tumor clearance (i.e., cold tumor). In this regard, epigenetic treatment of DNMT or EZH2 inhibitors (i.e., epi-drugs that remove DNA methylation and H3K27me3), alone or in combination, have been shown to restore CD8+ T cell infiltration and the sensitivity to ICB [2,4].

In the Special Issue of the journal *Epigenomes*, Lee et al. summarized several recent findings that epi-drugs were found to have immunomodulatory properties [5]. For example, treatment with azacytidine, a DNA methyltransferase inhibitor (DNMTi), induced the expression of genes involved in innate immunity, antigen presentation, and interferon signaling in cancer [6,7], while treatment with histone deacetylase (HDAC) inhibitor promoted the activation and secretion of inflammatory cytokines for effector T cells to induce other inflammatory cytokines [8,9]. Inhibition of histone lysine-specific demethylase LSD1 also reprogrammed tumor-associated macrophages into M1-like macrophages by increased methylation on H3K4 and H3K9 [10]. Furthermore, combination treatment of DNMT and EZH2 inhibitor improved immunotherapy by the reactivation of Th1-type chemokines, allowing T-cell trafficking in the tumor microenvironment [4].

It must be noted that more than 50% of the human genome is made of transposable elements (TEs), which are highly repetitive DNA sequences [11]. The TEs have lost their ability to transpose due to acquired mutation and/or epigenetic silencing. In a review article, the author stated that treatment with epi-drugs, such as DNMTi, may reactivate the transcription of TEs, leading to the formation of long double-stranded RNA (dsRNA). Accumulation of dsRNA can activate antiviral signaling pathways, and ultimately results in the secretion of type I and III interferons (IFNs), antiviral response and chemotaxis of immune cells [12].

In this regard, several clinical trials involving combination treatment of epi-drugs and ICB are underway in several human cancers, including breast cancer [5,13]. Epigenetic drugs might offer combinational opportunities with immune checkpoint inhibitors in cancer treatment, leading to synergistic antitumor effects. The role of epigenetic gene silencing in the homeostasis of immune cells and tumor cells needs further investigation, and will lead to more effective epigenetic drug and immunotherapy cotreatment.

## Figures and Tables

**Figure 1:**
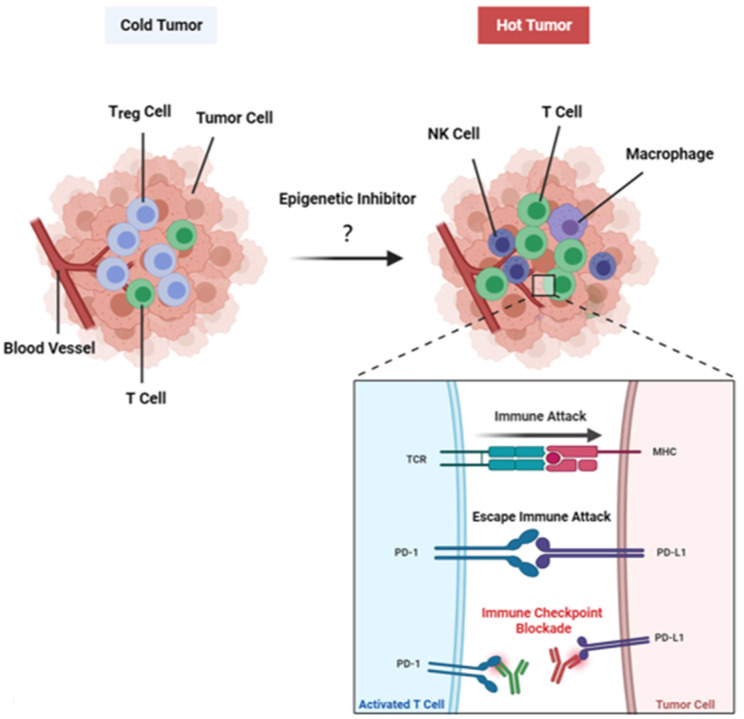
Tumors may escape immunosurveillance by epigenetic silenicng of immune-related genes (for example, Th1-type chemokine), leading to a non-inflammed “cold” tumor. Treatment with epigenetic inhibitors (epi-drugs) restores the expression of those immune-related genes, resulting in the infiltration of immunocytes (i.e., hot tumor) and restoring the sensitivity of the tumor cells to immune checkpoint blockade.
